# Transcriptome analysis reveals the molecular mechanisms underlying the enhancement of salt-tolerance in *Melia azedarach* under salinity stress

**DOI:** 10.1038/s41598-024-61907-5

**Published:** 2024-05-14

**Authors:** Na Li, Tianyun Shao, Li Xu, Xiaohua Long, Zed Rengel, Yu Zhang

**Affiliations:** 1 College of Resources and Environmental Sciences, Nanjing Agricultural University, Nanjing, 210095 China; 2 Institute of Crop sciences, Inner Mongolia Academy of Agricultural & Animal Husbandry Sciences, Inner Mongolia, 010031 China; 3 Soil Science and Plant Nutrition, UWA School of Agriculture and Environment, The University of Western Australia, 35 Stirling Highway, Perth, WA 6009 Australia; 4 Institute for Adriatic Crops and Karst Reclamation, 21000 Split, Croatia

**Keywords:** Salt stress, *Melia azedarach*, Transcriptome, Plant hormone signaling pathways, MYC2, Ecology, Plant sciences, Ecology, Environmental sciences

## Abstract

*Melia azedarach* demonstrates strong salt tolerance and thrives in harsh saline soil conditions, but the underlying mechanisms are poorly understood. In this study, we analyzed gene expression under low, medium, and high salinity conditions to gain a deeper understanding of adaptation mechanisms of *M. azedarach* under salt stress. The GO (gene ontology) analysis unveiled a prominent trend: as salt stress intensified, a greater number of differentially expressed genes (DEGs) became enriched in categories related to metabolic processes, catalytic activities, and membrane components. Through the analysis of the category GO:0009651 (response to salt stress), we identified four key candidate genes (*CBL7*, *SAPK10*, *EDL3*, and *AKT1*) that play a pivotal role in salt stress responses. Furthermore, the KEGG (Kyoto Encyclopedia of Genes and Genomes) pathway enrichment analysis revealed that DEGs were significantly enriched in the plant hormone signaling pathways and starch and sucrose metabolism under both medium and high salt exposure in comparison to low salt conditions. Notably, genes involved in JAZ and MYC2 in the jasmonic acid (JA) metabolic pathway were markedly upregulated in response to high salt stress. This study offers valuable insights into the molecular mechanisms underlying *M. azedarach* salt tolerance and identifies potential candidate genes for enhancing salt tolerance in *M. azedarach*.

## Introduction

Saline soils exhibit have very different properties compared to normal soils, including increased salinity, reduced organic matter content, and impaired structural stability^1^. Soil salinization has become a global concern as it reduces plant growth and limits land use efficiency^2^. Soil salinization impacts about one-third of the world's irrigated land and decreases agricultural yield by approximately 22%^3^.

The main harmful effects of soil salinity on plants typically include osmotic stress, ionic imbalance, oxidative injury from reactive oxygen species (ROS), etc^4^. Plants trigger a series of transcriptional modifications upon detection of excessive soil salinity with the aim of synthesizing defensive proteins and metabolites^5,6^. Notably, genes associated with plant salt tolerance are involved in a multitude of physiological pathways, encompassing signal transduction, secondary metabolism, the management of reactive oxygen species, and transcriptional regulation, etc^7,8^.

*Melia azedarach* is a valuable afforestation tree species characterized by its rapid growth, remarkable adaptability, and notable tolerance to abiotic stresses^9,10^. It is also considered one of the most valuable medicinal plants due to its antiviral, antibacterial, and insecticidal properties^11^. The cultivation of *M. azedarach* on saline soil can serve as a means of ameliorating saline soils and providing economic benefits through plant products^12,13^.

Changes in the expression of plant genes under different salinity stress conditions can be quantified by RNA sequencing technology^14^, which plays a key role in elucidating the biochemical, physiological, and molecular mechanisms of plant responses to salt stress^15^. However, transcriptome analysis of *M. azedarach* under different levels of salt stress has not been found in available reports.

The study investigated the adaptation mechanisms of *M. azedarach* to salt stress by comparative analysis of transcriptome changes in *M. azedarach* roots under low, medium, and high salinity environments. This study will lay the foundation for elucidating the adaptation mechanisms of *M. azedarach* to a various abiotic stresses.

## Materials and methods

### Field set-up

The field experiment was conducted in the Xuwei New District (34° 37′ N, 119° 29′ E) within Lianyungang City, Jiangsu Province. This area is distinguished by a warm temperate humid monsoon maritime climate, displaying characteristics of both warm temperate and northern subtropical climates. The average annual temperature in the region is 14 °C, accompanied by an annual average rainfall of 901 mm and an annual average evaporation of 855 mm.

The soil salinity is predominantly composed of sodium chloride and belongs to the category of silty coastal saline soil. The experimental site was partitioned into three distinct plots characterized by differing levels of salinity: low salinity (L, 148.4 μS/cm, 0.37 g/kg), medium salinity (M, 2828 μS/cm, 7.52 g/kg), and high salinity (H, 4326 μS/cm, 11.5 g/kg). The electrical conductivity of the experimental sites with low, medium, and high salinity levels were respectively 148.4, 2828, and 4326 μS/cm. The sodium ion contents for these levels were 0.26, 3.68, and 4.50 g/kg respectively. *Melia azedarach* was planted with 2.0 m of intra-row and 3.0 m of inter-row distance. *M. azedarach* was irrigated once a week during the first month of planting and no irrigation was done for the rest of the year.

### Sample collection

In August 2020 (after 40 months of *M. azedarach* growth), three samples of *M. azedarach* roots were collected from each plot. The *M. azedarach* roots were excavated with a shovel and subsequently stored in a − 80 °C freezer for transcriptome analysis. Root samples (R) collected for transcriptome analysis were labelled: HR, MR, and LR for high, medium, and low salinity, respectively.

### *M. azedarach* transcriptomics

#### RNA extraction, library construction, and RNA-Seq

The RNA was extracted from the roots of *M. azedarach* grown in soils characterized by high, medium, and low salt content using the Trizol reagent kit (Invitrogen, Carlsbad, California, USA)^16^. The quality of the RNA samples was checked using a NanoDrop 2000 Spectrophotometer. A260/280 was in the range of 1.80–2.0. A total of 1 μg of RNA per sample served as the input material for sequencing. Biomarker Technologies, based in Beijing, China, performed the RNA sequencing. Sequencing libraries were constructed using a NEBNext®Ultra™ RNA Library Prep Kit for Illumina^®^ (NEB, MA, USA), following the manufacturer's prescribed procedures. Index codes were incorporated to distinguish sequences associated with each specific sample. Library quality was assessed using an Agilent Bioanalyzer 2100 system. The sequencing took place on an Illumina Hiseq 2000 platform, generating paired-end reads.

To obtain clean data, reads containing adapters, reads containing poly-N sequences, and low-quality reads were removed from the raw data^17^. Additionally, the Q30 score and the GC content of the clean reads were calculated. All subsequent analyses were conducted using these high-quality clean reads.

#### Transcriptome assembly and function annotation

Transcriptome assembly was accomplished based on the left.fq and right.fq using Trinity with min_kmer_cov and all other parameters set to default^18^. Gene function was annotated based on the following databases: NR (NCBI non-redundant protein sequences), Pfam (Protein family), KEGG (Kyoto Encyclopedia of Genes and Genomes), and GO (Gene Ontology).

#### Differentially expressed genes (DEGs)

Differential expression analysis was performed using the DESeq R package (v. 1.10.1). Genes with | log_2_ (foldchange) |≥ 1 & an adjusted *p*-value < 0.05 found by DESeq were assigned as differentially expressed. The GO enrichment analysis of DEGs was implemented by the topGO R packages based on Kolmogorov–Smirnov test. KOBAS software was used to test the statistical enrichment of DEGs in KEGG pathways^19^.

#### RT-qPCR validation of DEGs

Total RNA was reverse-transcribed into cDNA using a TaKaRa reverse transcription kit (TaKaRa, 6210A). To verify the reliability of RNA-Seq data, 20 DEGs with high expression levels were randomly selected for RT-qPCR validation using ABI Stepone Plus Real-Time PCR System with Tower (ABI, US). Premier 6.0 was utilized for primer design, with the actin gene serving as the internal reference gene. The PCR cycling parameters were initial 95 °C for 2 min, then 40 cycles of 95 °C for 5 s, and 60 °C for 15 s. The experimental data were analyzed by the 2^−ΔΔCt^ method.

### Statistics and data nalysis

An independent sample *t*-test was performed using IBM SPSS Statistics 20 software (IBM, Armonk, New York, USA), and the significance level of the difference was determined based on *p* < 0.05. Simulation diagram, bar graphs, bubble charts, and curvilinear regression were constructed by R package gglot 2 (v. 3.2.0). The Principal Component Analysis (PCA) was done by R package vegan (v. 2.5.5). Heatmap was constructed by R package heatmap (v. 1.0.8). Venn diagrams were generated using the R package venn diagram (v. 1.6.20). The GO classification of DEGs was generated using GraphPad Prism (v. 8.0.1). The RNA-seq and RT-qPCR data of 20 selected DEGs were compared using Origin 2022.

## Results

### Transcriptome sequencing quality analysis

From the nine transcriptome libraries, a total of 54.88 Gb of clean reads were obtained, with average sample contributing approximately 6.1 Gb of clean data. It is noteworthy that more than 82% of the sequences within each library were successfully mapped, which underscores the suitability of the assembled transcriptome for conducting differential expression analyses. Furthermore, the sequencing data displayed a high level of quality, with a Q30 base percentage exceeding 93.5% (Table S1).

The saturation analysis of the transcriptome data exhibited a saturable curve. This pattern suggests that the quantity of effective sequencing data obtained was sufficient for the study objectives (Fig. S1).

### DEGs analysis of *M. azedarach* under salt stress

**Figure 1:**
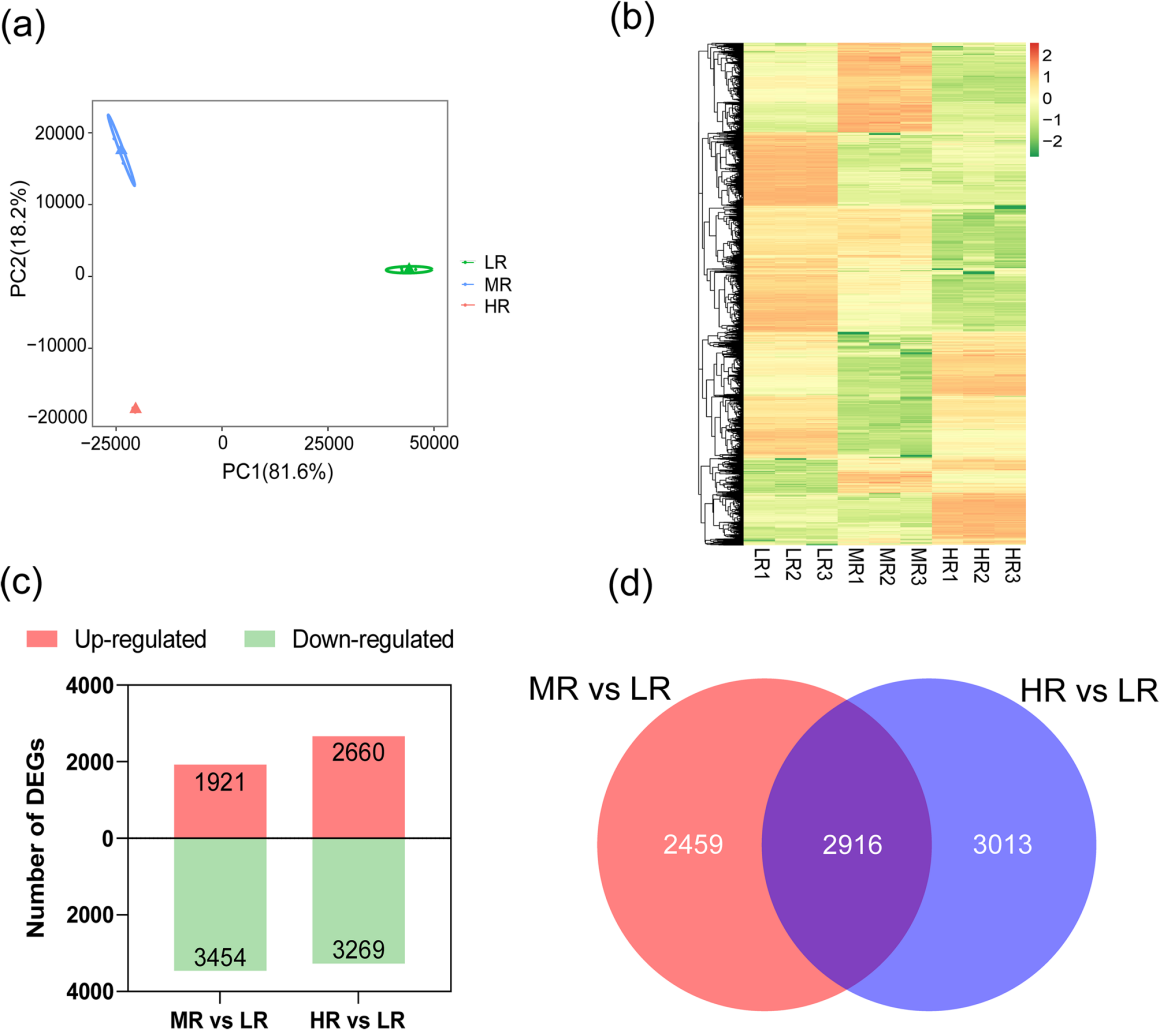
DEGs of *Melia azedarach* under salt stress. (**a**) Principal component analysis based on DEGs. (**b**) Heatmap analysis of DEGs. (**c**) Bar graph of up-regulated and down-regulated DEGs. (**d**) Venn diagram of DEGs. *LR* roots in low salinity soil, *MR* roots in medium salinity soil, *HR* roots in high salinity soil. Biological replicates are indicated by the numbers 1–3.

The complete list of DEGs is given in the Supplementary Data (Table S2). The results of PCA indicate that samples from different salinities were distinctly separated from one another, meaning that the transcriptome of *M. azedarach* roots exhibited substantial variations in response to different salinity levels (Fig. 1a). The heatmap analysis demonstrated that the patterns of up-regulation and down-regulation of DEGs varied significantly under different salinity conditions (Fig. 1b). With increasing salt stress, there was an apparent trend of a growing number of up-regulated DEGs, accompanied by a corresponding decline in the number of down-regulated DEGs (Fig. 1c). The Venn diagram showed that 2916 DEGs were shared in the MR vs. LR and the HR vs. LR comparisons (Fig. 1d).

**Figure 2:**
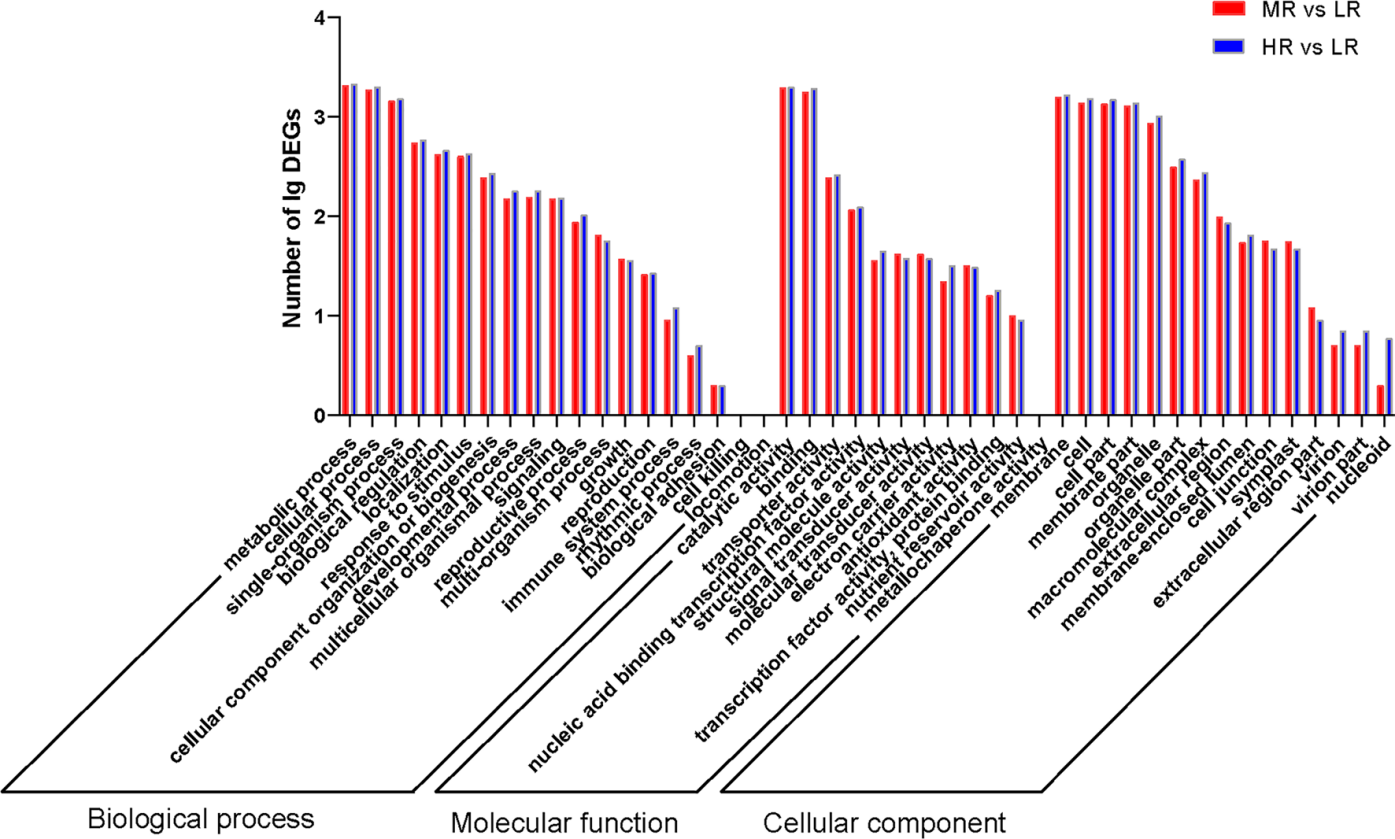
GO classification of DEGs. *LR* roots in low salinity soil, *MR* roots in medium salinity soil, *HR* roots in high salinity soil.

The GO functional enrichment analysis of DEGs revealed their enrichment in 47 GO annotations. Among these, the biological process (BP) category accounted for 39.62%, the cellular component (CC) category accounted for 38.35%, and the molecular function (MF) category accounted for 22.03% of the enriched terms. Within the BP category, the DEGs were predominantly enriched for terms such as metabolic process (GO:0008152), cellular process (GO:0009987), and single organism process (GO:0044699). In the CC category, the primary enrichments included membrane (GO:0016020), cell (GO:0005623), and cell part (GO:0044464). As the severity of salt stress increased, an increasing number of DEGs was enriched in the GO annotation (Fig. 2).

The enrichment data pertaining to up-regulated DEGs within the 47 GO annotations, as delineated in Table S3, exhibited a consistent pattern. This pattern revealed a notable increase in the number of up-regulated DEGs as salt stress was exacerbated. This trend was particularly prominent in categories associated with metabolic processes, catalytic activities, and membrane.

**Figure 3:**
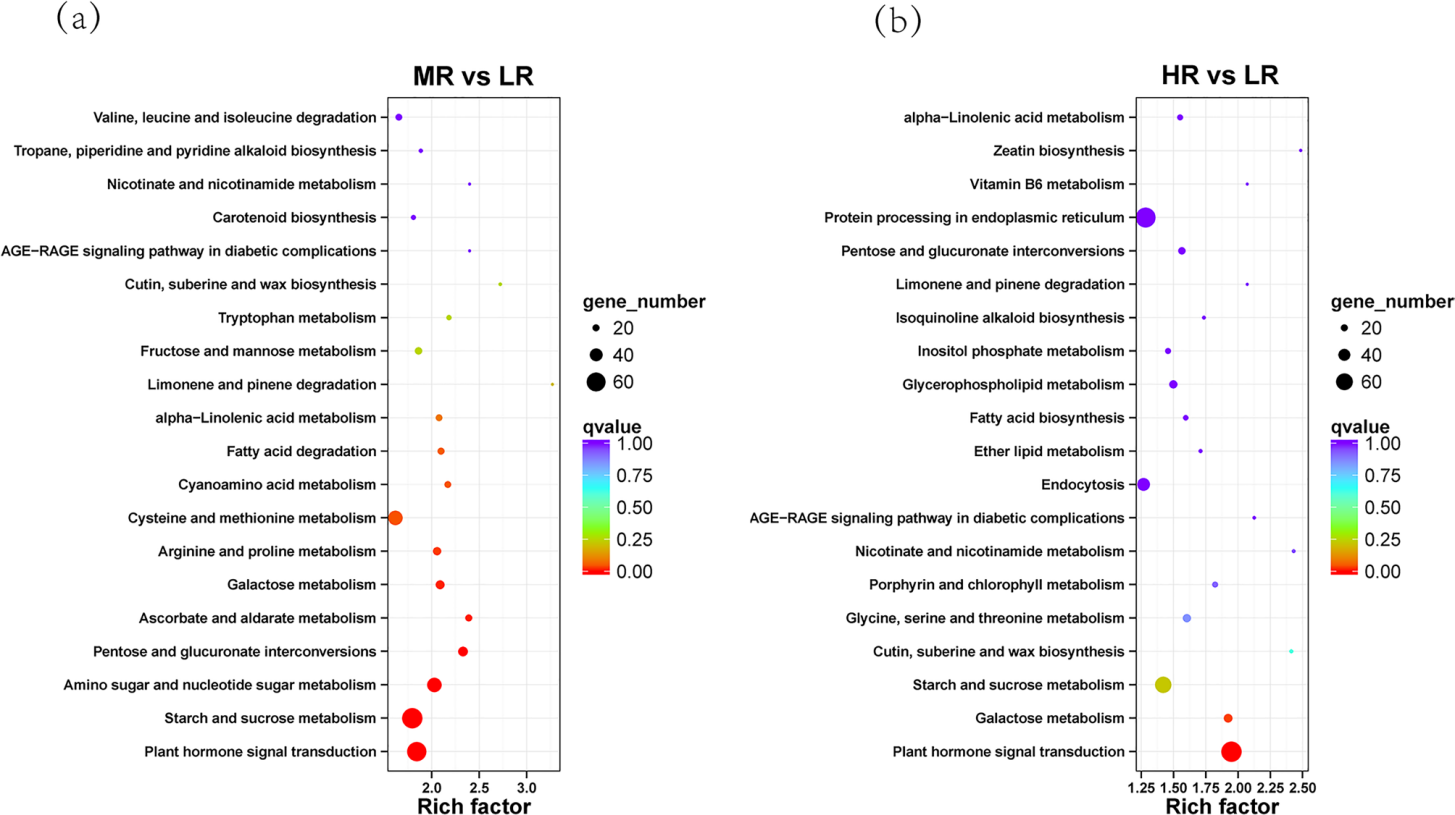
Bubble chart of DEGs enrichment in KEGG pathways. Each circle in the figure represents a KEGG pathway, and the color of the circle represents the *q*-value. The smaller the value, the more reliable the enrichment significance of DEGs in this pathway. The size of the circle indicates the number of genes enriched in the pathway. The larger the circle, the more genes. *LR* roots in low salinity soil, *MR* roots in medium salinity soil, *HR* roots in high salinity soil.

To identify the metabolic pathways involved in the response to salt stress, we conducted the KEGG pathways enrichment analysis. It revealed a significant enrichment of DEGs in the MR vs. LR in starch and sucrose metabolism, plant hormone signal transduction, amino sugar and nucleotide sugar metabolism, as well as in cysteine and methionine metabolism (Fig. 3a). In the HR vs. LR comparison, there was a notable enrichment in pathways related to plant hormone signal transduction and starch and sucrose metabolism (Fig. 3b).

**Figure 4:**
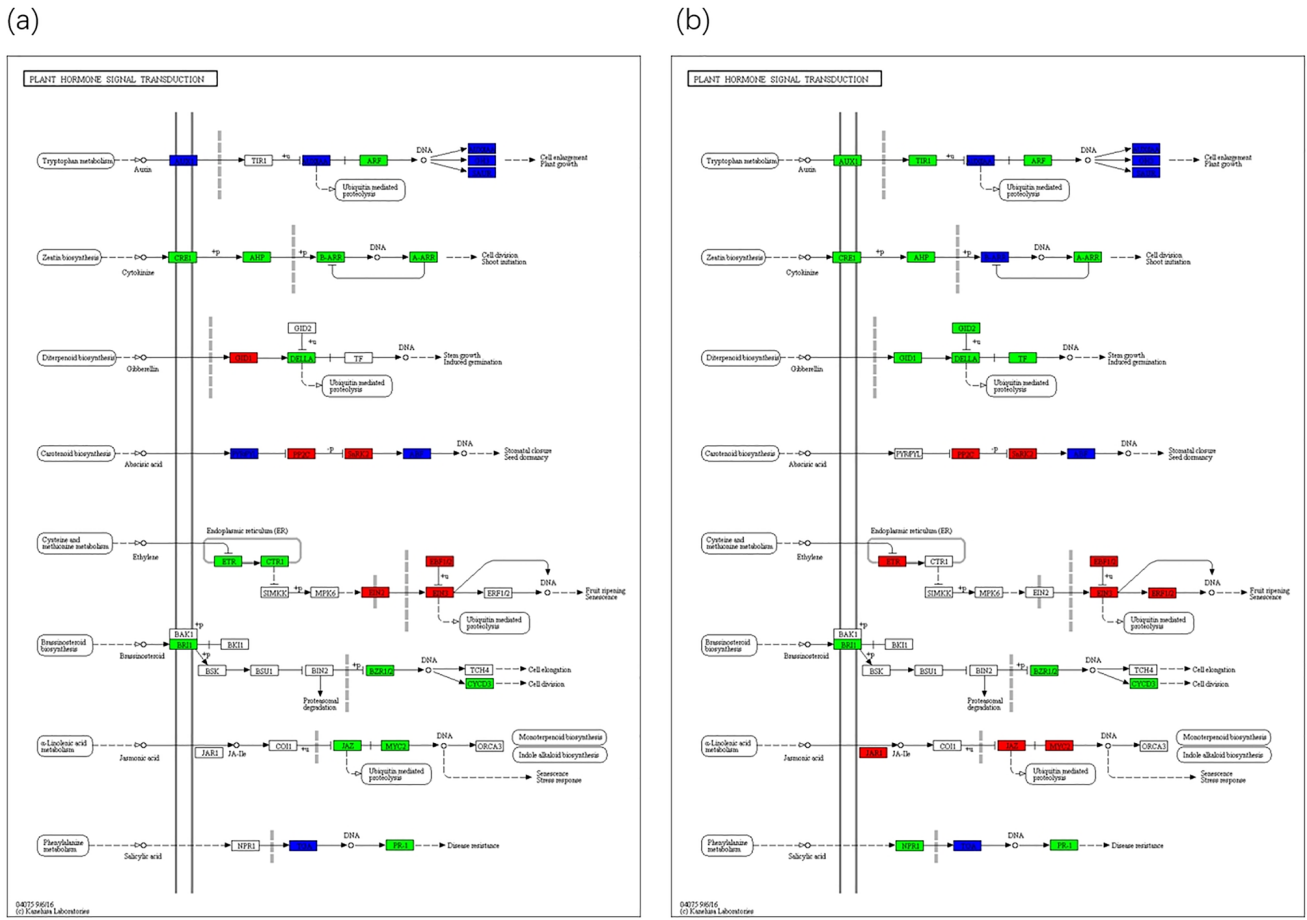
DEGs in plant hormone signaling pathways. (**a**) MR vs. LR (**b**) HR vs. LR. The light green background in the figure indicates the genes existing in the KEGG pathway of the species genome; red background indicates up-regulated DEGs involved in the pathway, and green indicates down-regulated DEGs involved in the pathway, blue indicates the pathway involving both up-regulated and down-regulated DEGs.

The signal transduction pathways associated with plant hormones (ko04075) is presented in Fig. 4. At varying severities of salt stress, there was a selective enrichment of *M. azedarach* genes involved in various phytohormonal signaling pathways, including auxin, cytokinin, gibberellin, abscisic acid, ethylene, brassinosteroid, jasmonic acid, and salicylic acid. Within brassinosteroid metabolism, the genes encoding BRI1, BZR1/2, and CYCD3 were downregulated in both MR vs LR and HR vs LR comparisons. This downregulation would be expected to result in the inhibition of cell elongation and cell division processes. Regarding jasmonic acid metabolism, genes encoding JAZ and MYC2 were upregulated in HR vs LR comparisons (Fig. 4b).

In response to salt stress and based on the GO analysis of BP under the term GO:0009651 (response to salt stress), four key DEGs were identified. The *CBL7* and *SAPK10* genes were up-regulated in MR vs. LR, the *EDL3* gene was up-regulated in HR vs. LR, and the *AKT1* gene was up-regulated in both MR vs. LR and HR vs. LR (Table 1).

**Table 1:** Key *M. azedarach* DEGs for adaptation to salt stress.

#ID	Name	MR vs. LR	HR vs. LR
c30606.graph_c0	*CBL7*	Up	Unchanged
c32333.graph_c0	*SAPK10*	Up	Unchanged
c37857.graph_c0	*EDL3*	Unchanged	Up
c36456.graph_c0	*AKT1*	Up	Up

### RT-qPCR

To assess the reliability of the RNA-Seq data, we randomly selected 20 DEGs with high expression levels for validation using RT-qPCR. The RT-qPCR primer sequences can be found in Table S4.

**Figure 5:**
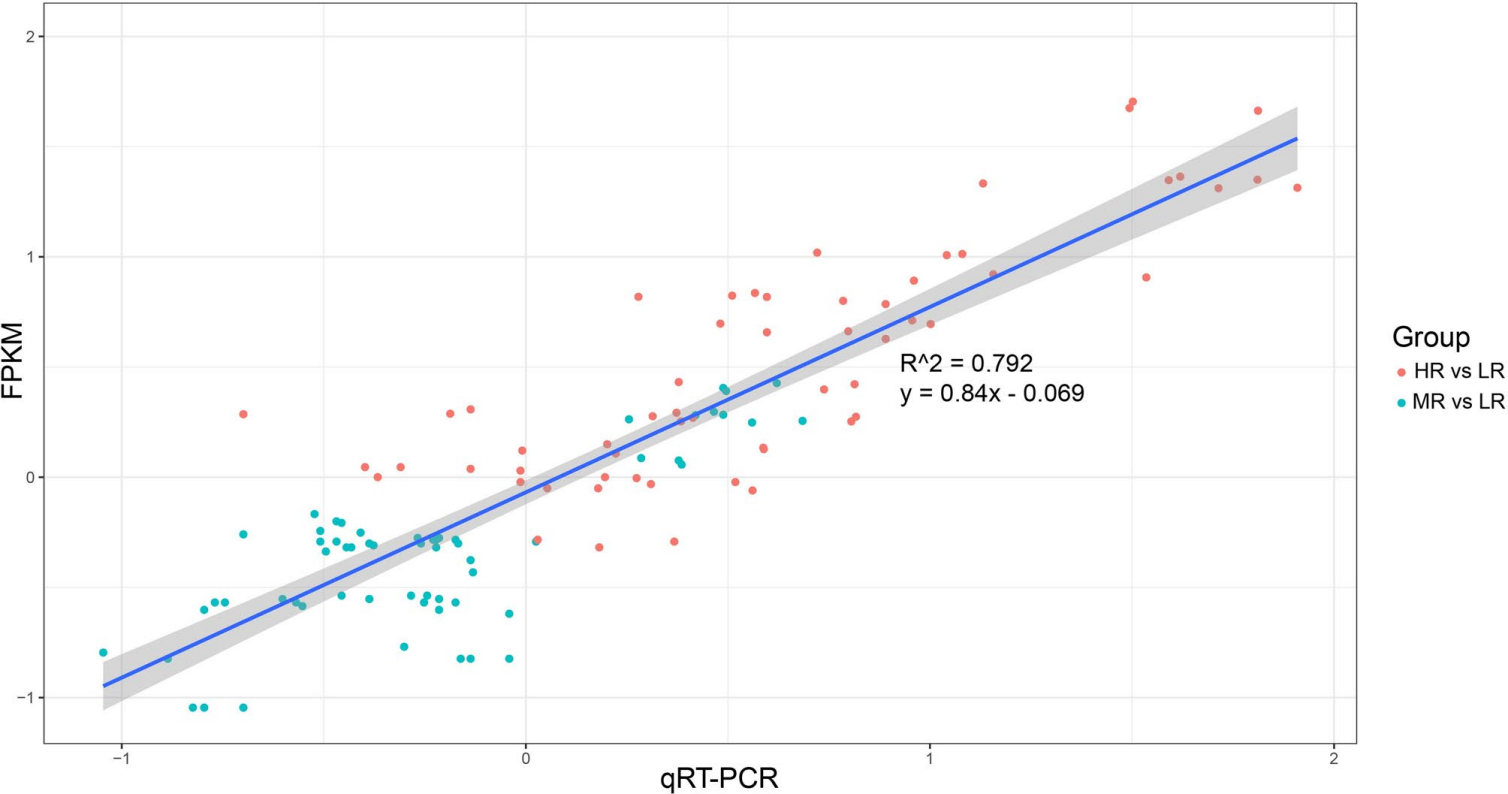
Linear regression of RT-qPCR and FPKM. *LR* roots in low salinity soil, *MR* roots in medium salinity soil, *HR* roots in high salinity soil.

A comparison between the RT-qPCR data for these 20 DEGs and the RNA-Seq results showed that a substantial proportion of the DEGs displayed consistent expression patterns. This congruence between the two datasets (Fig. S2) affirmed the reliability of the RNA-Seq data. The comprehensive curvilinear regression analysis conducted on the 20 DEGs unveiled a notable correlation coefficient of R^2^ = 0.792 between the RT-qPCR and FPKM data (Fig. 5). This robust correlation coefficient further confirmed the reliability of the RNA-Seq data and their suitability for this study.

## Discussion

The excessive accumulation of Na^+^ in the soil can result in soil compaction, hindered drainage, detrimental impacts on soil health, and ultimately soil degradation^20^. Organic matter and alkaline phosphatase activity play pivotal roles in the global cycles of carbon and phosphorus, respectively^12^. In our previous research, it was found that *M. azedarach* could significantly decrease Na^+^ content, increase organic matter content, and enhance alkaline phosphatase activity in rhizosphere soil^21^. These findings underscore the positive influence of *M. azedarach* cultivation in improving soil health and facilitating efficient nutrient cycling within the soil ecosystem.

Similarly, salt stress also exerts a pronounced adverse effect on plant physiology. On the one hand, salt stress-induced osmotic differentials lead to a blockage of water uptake from the soil by the root system, which results in water-loss wilting and even death of the plant^22^. On the other hand, salt stress triggers the accumulation of reactive oxygen species (ROS) in plants, thereby instigating detrimental processes such as lipid peroxidation, membrane degradation, and damage to DNA and proteins^23,24^. Plant membranes, as a key biological barrier, protect cells and organelles from the harmful effects of salt stress^25^, and the stability and function of membrane structural integrity are the basis of normal cell metabolism and overall physiology^26^. Under unfavorable conditions such as salt stress, plants can alter the content and composition of membrane lipids to adapt to the effects of adverse factors^27^. The GO analysis revealed that DEGs were increasingly associated with *M. azedarach* membranes as the severity of salt stress increased (Fig. 2), which corroborates the above arguments.

The KEGG pathway enrichment analysis undertaken in this investigation revealed that, in comparison to low salinity conditions, DEGs under medium- and high-salinity stress exhibited substantial enrichment in pathways associated with plant hormone signal transduction (Fig. 3a,b). Notably, jasmonic acid (JA) emerged as a pivotal stress-responsive hormone in plants, effectively safeguarding and sustaining the integrity of plant cells under a diverse array of stresses by elevating the expression of antioxidant defense mechanisms^28,29^. Within the JA pathway, MYC2 is a pivotal transcription factor, governing responses to a multitude of biotic and abiotic stresses^30^. When grown in highly saline soil, *M. azedarach* demonstrated an enhanced capacity to counteract oxidative stress by upregulating genes related to MYC2 (Fig. 4b).

In the GO analysis, within the category denoted as GO:0009651 – response to salt stress, four key DEGs were discerned to be up-regulated as soil salinity increased, namely *CBL7*, *SAPK10*, *EDL3*, and *AKT1* (Table 1). Calcium signaling is a key determinant of plant salt tolerance, and *CBL7* is a unique set of calcium sensors in plants^31,32^. Meanwhile, *SAPK10* and *EDL3* genes regulate the ability of plants to adapt to salt stress by modulating abscisic acid-induced antioxidant defense mechanisms^33,34^. The up-regulation of *CBL7, SAPK10* and *EDL3* genes represents the enhancement of salt tolerance in plants. Similarly, AKT1 plays a crucial role in maintaining K^+^ and Na^+^ homeostasis in plants, and its up-regulation also enhances plant salt tolerance^35^.

## Conclusion

The cultivation of *M. azedarach* may be an effective strategy for improving the properties of saline soil. With increasing soil salinity, *M. azedarach* had a large number of DEGs within cell membrane components, thus reinforcing the role of the cell membrane as a biological barrier, safeguarding the cytosol and organelles from the detrimental impacts of salt stress. Additionally, *M. azedarach* upregulated the expression of MYC2 in roots, a pivotal transcription factor within the JA hormone pathway, which could enhance tolerance to oxidative stress. This study identified four crucial DEGs associated with the "salt stress response" including *CBL7*, *SAPK10*, *EDL3*, and *AKT1*. Collectively, these discoveries provide significant insights into the mechanisms through which *M. azedarach* adapts to salt stress.

### Supplementary Information

Supplementary Figure S1.

Supplementary Figure S2.

Supplementary Table S1.

Supplementary Table S2.

Supplementary Table S3.

Supplementary Table S4.

Supplementary Legends.

## Data Availability

The datasets used and/or analysed during the current study available from the corresponding author on reasonable request.
