# Supplementary material for: Transcriptome analysis reveals the molecular mechanisms underlying the enhancement of salt-tolerance in Melia azedarach under salinity stress
Source: Sci Rep. 2024 May 14;14:10981. doi: 10.1038/s41598-024-61907-5 (PMC11094156; doi:10.1038/s41598-024-61907-5)
Supplement: Supplementary file 1 — Supplementary Figure S1. [file 41598_2024_61907_MOESM1_ESM.docx]

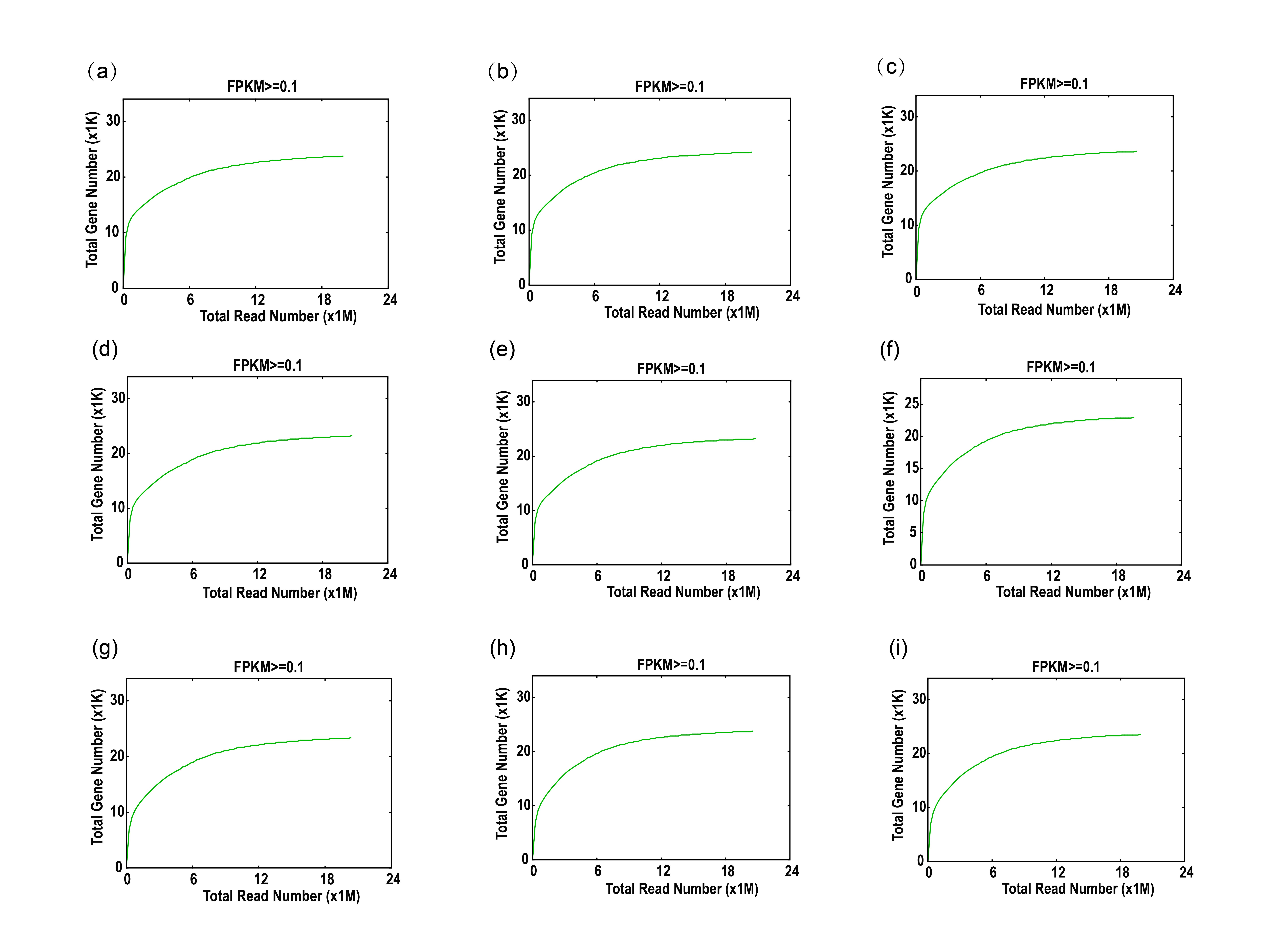


Figure S1 Simulation diagram of transcriptome sequencing data saturation, including samples LR1 (a), LR2 (b), LR3 (c), MR1 (d), MR2 (e), MR3 (f), HR1 (g), HR2 (h), and HR3 (i). Note: the saturation curve is drawn by dividing mapped reads into 100 equal parts, gradually increasing the number of genes detected by data viewing. The abscissa is the number of reads (in 10^6^), and the ordinate is the number of genes detected (in 10^3^). LR- roots in low salinity soil, MR- roots in medium salinity soil, HR- roots in high salinity soil. Biological replicates are indicated by the numbers 1-3.
