# Supplementary figures and images for: Transcriptome analysis reveals the molecular mechanisms underlying the enhancement of salt-tolerance in Melia azedarach under salinity stress
Source: Sci Rep. 2024 May 14;14:10981. doi: 10.1038/s41598-024-61907-5 (PMC11094156; doi:10.1038/s41598-024-61907-5)

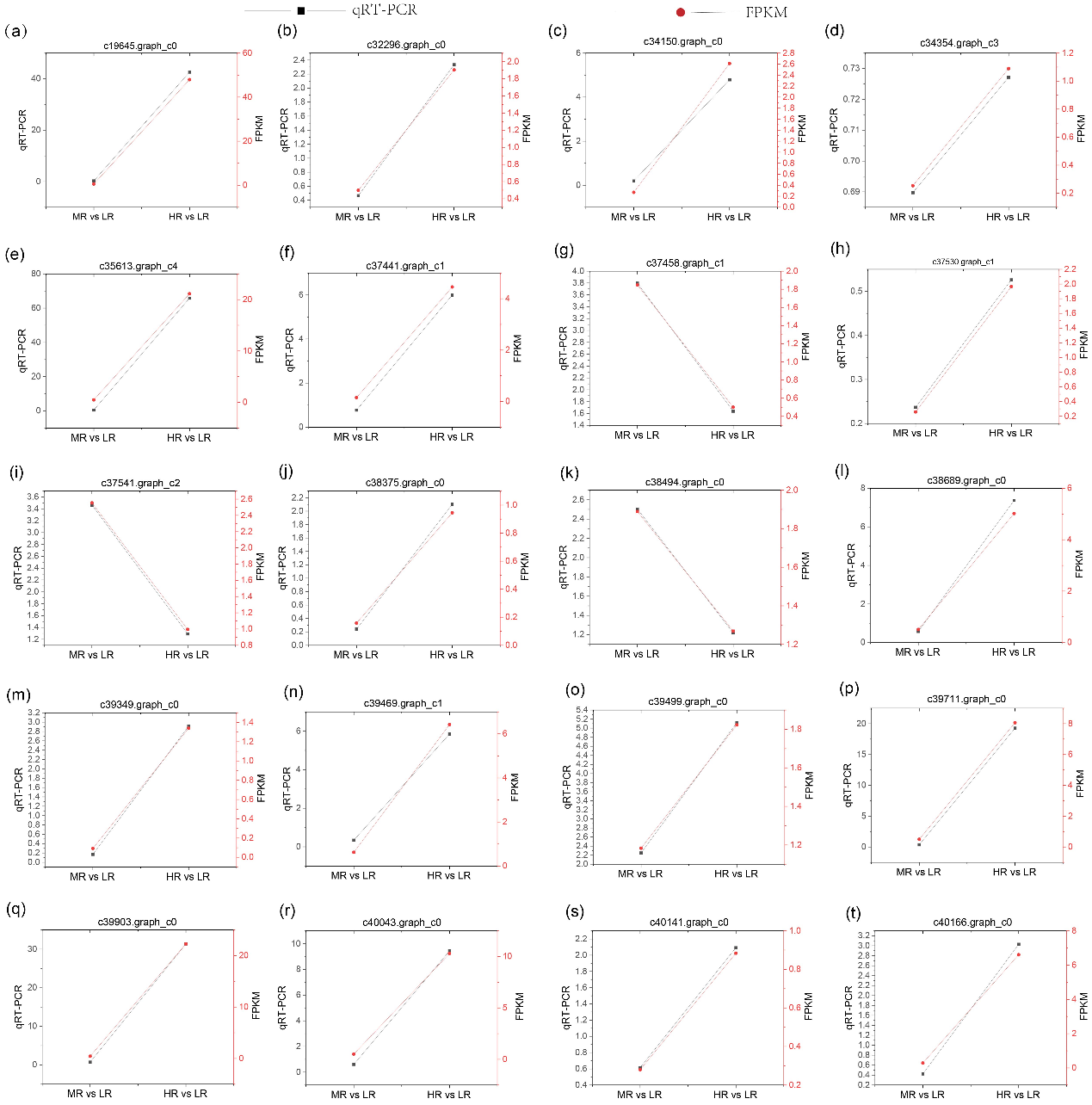


Figure S2 Comparison between RT-qPCR data and RNA-seq data of 20 DEGs.

Supplement: Supplementary file 2 — Supplementary Figure S2. [file 41598_2024_61907_MOESM2_ESM.docx]
