# Supplementary material for: Transcriptome analysis reveals the molecular mechanisms underlying the enhancement of salt-tolerance in Melia azedarach under salinity stress
Source: Sci Rep. 2024 May 14;14:10981. doi: 10.1038/s41598-024-61907-5 (PMC11094156; doi:10.1038/s41598-024-61907-5)
Supplement: Supplementary file 3 — Supplementary Table S1. [file 41598_2024_61907_MOESM3_ESM.docx]

TABLE S1 Evaluation statistics of transcriptome sequencing data of *M. azedarach*

| Sample | Clean reads | Clean bases | Mapped reads | GC content (%) | ≥Q30 (%) |
| --- | --- | --- | --- | --- | --- |
| LR1 | 19,906,295 | 5.95Gb | 16541879 (83.10%) | 44.97 | 94.26 |
| LR2 | 20,430,005 | 6.11Gb | 16999191 (83.21%) | 45.09 | 94.11 |
| LR3 | 21,413,523 | 6.40Gb | 17895471 (83.57%) | 44.97 | 94.08 |
| MR1 | 20,673,950 | 6.19Gb | 17468030 (84.49%) | 45.28 | 93.52 |
| MR2 | 20,703,389 | 6.19Gb | 17449984 (84.29%) | 45.25 | 94.02 |
| MR3 | 19,588,748 | 5.87Gb | 16762383 (85.57%) | 45.62 | 93.97 |
| HR1 | 20,337,358 | 6.09Gb | 16773095 (82.47%) | 44.82 | 94.14 |
| HR2 | 20,533,706 | 6.15Gb | 16879561 (82.20%) | 44.59 | 93.81 |
| HR3 | 19,878,532 | 5.95Gb | 16311438 (82.06%) | 44.64 | 93.92 |

Note: Clean reads: filtered reads; Clean bases: clean reads multiplied by their lengths; Mapped reads: filtered reads compared to transcript or UniGene; GC content (%): the percentage of G and C bases in the total bases in reads; ≥Q30 (%): the percentage of bases whose median value of filtered reads is greater than or equal to 30. LR: roots in low salinity soil, MR: roots in medium salinity soil, HR: roots in high salinity soil. Biological replicates are indicated by the numbers 1-3.
