# Supplementary material for: Transcriptome analysis reveals the molecular mechanisms underlying the enhancement of salt-tolerance in Melia azedarach under salinity stress
Source: Sci Rep. 2024 May 14;14:10981. doi: 10.1038/s41598-024-61907-5 (PMC11094156; doi:10.1038/s41598-024-61907-5)
Supplement: Supplementary file 5 — Supplementary Table S3. [file 41598_2024_61907_MOESM5_ESM.docx]

TABLE S3 Enrichment data associated with up-regulated DEGs in 47 GO annotations

| GO_classify1 | GO_classify2 | Up-regulated DEGs  (MR vs LR) | Up-regulated DEGs  (HR vs LR) |
| --- | --- | --- | --- |
| Cellular Component | cell | 487 | 696 |
| Cellular Component | membrane | 449 | 685 |
| Cellular Component | cell part | 481 | 684 |
| Cellular Component | membrane part | 360 | 572 |
| Cellular Component | organelle | 328 | 471 |
| Cellular Component | organelle part | 124 | 184 |
| Cellular Component | macromolecular complex | 107 | 125 |
| Cellular Component | membrane-enclosed lumen | 33 | 37 |
| Cellular Component | extracellular region | 15 | 22 |
| Cellular Component | cell junction | 13 | 19 |
| Cellular Component | symplast | 13 | 19 |
| Cellular Component | virion | 5 | 6 |
| Cellular Component | virion part | 5 | 6 |
| Cellular Component | extracellular region part | 1 | 1 |
| Cellular Component | nucleoid | 1 | 0 |
| Molecular Function | binding | 606 | 828 |
| Molecular Function | catalytic activity | 555 | 799 |
| Molecular Function | transporter activity | 80 | 98 |
| Molecular Function | nucleic acid binding transcription factor activity | 39 | 45 |
| Molecular Function | electron carrier activity | 10 | 21 |
| Molecular Function | structural molecule activity | 14 | 20 |
| Molecular Function | signal transducer activity | 7 | 17 |
| Molecular Function | molecular transducer activity | 7 | 17 |
| Molecular Function | antioxidant activity | 7 | 11 |
| Molecular Function | transcription factor activity, protein binding | 9 | 6 |
| Molecular Function | nutrient reservoir activity | 3 | 4 |
| Molecular Function | metallochaperone activity | 1 | 1 |
| Molecular Function | protein tag | 0 | 1 |
| Biological Process | metabolic process | 652 | 881 |
| Biological Process | cellular process | 589 | 844 |
| Biological Process | single-organism process | 438 | 642 |
| Biological Process | biological regulation | 200 | 263 |
| Biological Process | localization | 137 | 215 |
| Biological Process | response to stimulus | 131 | 194 |
| Biological Process | cellular component organization or biogenesis | 83 | 113 |
| Biological Process | multicellular organismal process | 58 | 73 |
| Biological Process | signaling | 49 | 72 |
| Biological Process | developmental process | 53 | 71 |
| Biological Process | reproductive process | 36 | 49 |
| Biological Process | multi-organism process | 17 | 30 |
| Biological Process | growth | 11 | 16 |
| Biological Process | reproduction | 8 | 11 |
| Biological Process | immune system process | 2 | 7 |
| Biological Process | rhythmic process | 3 | 4 |
| Biological Process | cell killing | 1 | 1 |
| Biological Process | biological adhesion | 0 | 0 |
| Biological Process | locomotion | 0 | 0 |
