# Supplementary material for: Transcriptome analysis reveals the molecular mechanisms underlying the enhancement of salt-tolerance in Melia azedarach under salinity stress
Source: Sci Rep. 2024 May 14;14:10981. doi: 10.1038/s41598-024-61907-5 (PMC11094156; doi:10.1038/s41598-024-61907-5)
Supplement: Supplementary file 6 — Supplementary Table S4. [file 41598_2024_61907_MOESM6_ESM.docx]

TABLE S4 Primers used for RT-qPCR to validate RNA-seq results.

| Gene ID | Gene Name | Forward Primer | Reverse Primer |
| --- | --- | --- | --- |
| c19645.graph_c0 | HSP21 | GTTCTACCTCTGCTGTTGT | ATGATGGAGACAATGGAGAG |
| c32296.graph_c0 | SAG20 | GAGTTGTCGGATGCTGAA | CTGTATGACGCCTTGAGAT |
| c34150.graph_c0 | BIP5 | CGACAGAAGGAAGAGTTGA | CACCGAAGAATACGATTGC |
| c34354.graph_c3 | METK4 | TTACTCGCACTCACTAACC | AGACCTGTAAGCAAGACTG |
| c35613.graph_c4 | MED37B | CGTTAAGCGTCTTATTGGAA | ACTAAGCATTGGTTCACTGA |
| c37441.graph_c1 | HSC-2 | ATTGGTGATGCCGCTAAG | TCTAAGAGAACAACGCTACA |
| c37458.graph_c1 | CAS1 | TCACATATTCCTATCCTCCATC | TTAGTTGCGTCTTCCTTGA |
| c37530.graph_c1 | UGD1 | CTGAACTGCGTCCAATAGA | TGTGGTGTTGAAGCGAAT |
| c37541.graph_c2 | ALDH2B4 | AACAGTTGCTTGCTTCTCT | GATGCCTCTTGCGGTATT |
| c38375.graph_c0 | LOX3.1 | CTTCTGCTTCTGCTCCTG | TTAGTCAGTGCCATCTCTTC |
| c38494.graph_c0 | RH8 | ATCAGGTGGAGGTATCTTCA | ATATGGTGTTGGTTGGCTAA |
| c38689.graph_c0 | HOP3 | TTGCTTCCTTATCCTCTTCC | AGAATCGCCGAACACATC |
| c39349.graph_c0 | LOX2.1 | TGGTAGTTGGTTCGGTAGA | GCATGGAAGTGGTCAGATT |
| c39469.graph_c1 | HSF30 | TAGCAACAGCATAACAACTC | GTCGCAACTCCTACAGATT |
| c39499.graph_c0 | EIN3 | TGACTACATCTATGCTTCCTC | CTGCTGCTTCCTCTTCTG |
| c39711.graph_c0 | HSP17.5-E | ATCTCCACCTTCACTTCCT | TCCTTCATCTTCCTCTTCAC |
| c39903.graph_c0 | HSP22.7 | TCGTCAAGATTGCTAATGC | ATCTCCACATCTCTTCCAAT |
| c40043.graph_c0 | FKBP62 | GCGAAGAAGGTAGTATAGTTG | GTCCACATCCTCCTCCAT |
| c40141.graph_c0 | XTH23 | ATTCGGCTTTCTTTCATCTG | GCTCGTTCTGTCCTCCTA |
| c40166.graph_c0 | NTR2 | TCAAGCAGGAACAGCATAA | CCAAGCAGACCTTCACTT |
| internal reference gene | Actin | AGGCATCCACGAGACCACTT | TGGCGCTAGAGCAGAAATTTC |
