# Supplementary material for: Transcriptome analysis reveals the molecular mechanisms underlying the enhancement of salt-tolerance in Melia azedarach under salinity stress
Source: Sci Rep. 2024 May 14;14:10981. doi: 10.1038/s41598-024-61907-5 (PMC11094156; doi:10.1038/s41598-024-61907-5)
Supplement: Supplementary file 7 — Supplementary Legends. [file 41598_2024_61907_MOESM7_ESM.docx]

# Supplementary figures and tables

Figure S1 Simulation diagram of transcriptome sequencing data saturation, including samples LR1 (a), LR2 (b), LR3 (c), MR1 (d), MR2 (e), MR3 (f), HR1 (g), HR2 (h), and HR3 (i). Note: the saturation curve is drawn by dividing mapped reads into 100 equal parts, gradually increasing the number of genes detected by data viewing. The abscissa is the number of reads (in 10^6^), and the ordinate is the number of genes detected (in 10^3^). LR- roots in low salinity soil, MR- roots in medium salinity soil, HR- roots in high salinity soil. Biological replicates are indicated by the numbers 1-3.

Figure S2 Comparison between RT-qPCR data and RNA-seq data of 20 DEGs.

Table S1 Evaluation statistics of transcriptome sequencing data of *M. azedarach*. Note: Clean reads: filtered reads; Clean bases: clean reads multiplied by their lengths; Mapped reads: filtered reads compared to transcript or UniGene; GC content (%): the percentage of G and C bases in the total bases in reads; ≥Q30 (%): the percentage of bases whose median value of filtered reads is greater than or equal to 30. LR: roots in low salinity soil, MR: roots in medium salinity soil, HR: roots in high salinity soil.

Table S2 The complete list of DEGs

Table S3 Enrichment data associated with up-regulated DEGs in 47 GO annotations

Table S4 Primers used for RT-qPCR to validate RNA-seq results.
